# Low preserved proximal femoral bone stock volume as a risk factor for periprosthetic femoral fractures. A study of 90 femurs

**DOI:** 10.3389/fsurg.2025.1659027

**Published:** 2025-10-14

**Authors:** Matic Kolar, Blaž Mavčič, Veronika Kralj-Iglič, Vane Antolič

**Affiliations:** 1 Department of Orthopaedic Surgery, University Medical Centre Ljubljana, Ljubljana, Slovenia; 2 Faculty of Medicine, Chair of Orthopaedics, University of Ljubljana, Ljubljana, Slovenia; 3 Laboratory of Clinical Biophysics, Faculty of Health Sciences, University of Ljubljana, Ljubljana, Slovenia

**Keywords:** hip, osteoarthritis, total hip arthroplasty, periprosthetic fractures, bone stock

## Abstract

**Background:**

Despite the longstanding awareness of the increasing incidence and consequences of periprosthetic proximal femoral fractures (PPFFs), and the rationale protective role of the preserved bone stock, no method for its evaluation, with the potential for routine clinical application, has been available. A novel method for the evaluation of preserved proximal femoral bone stock volume (*V*_PF_) in conventional primary total hip arthroplasty (THA) on routinely available hip radiographs was introduced and compared with clinical data.

**Methods:**

Study was designed according to the standard protocol for retrospective matched case-control research. 30 cases of late PPFFs (minimum 1 year postoperatively) were identified in the hospital database of all implanted Anatomic Benoist Girard (ABG) II femoral stems. For every case, 2 age-/sex-/implant size-/surgeon-matched controls were found. The *V*_PF_ was evaluated for each hip, and the mean values in both groups were compared. The accuracy and intra-/inter-rater reliability of the novel method were tested. Regression subanalyses were performed to identify factors influencing the risk of PPFFs, and to assess correlations between *V_PF_* and other covariables.

**Results:**

The mean *V_PF_* in the group of cases was 113.8 ± 21.0 cm^3^ and significantly lower compared to 164.0 ± 38.4 cm^3^ in the control group (*P* < 0.01). The method's reliability and accuracy were within good to excellent range. The *V*_PF_ was the sole significant factor influencing the risk of PPFFs (aOR = 0.92). The cut-off value was determined at 128.5 cm^3^. The regression analysis indicated an interplay of intuitively connected factors in the long-term PPFFs prognosis (*V*_PF_, stress shielding, subsidence).

**Conclusions:**

The presented results indicate that bone stock preservation (with *V_PF_* as a quantitative measure) is crucial for the prevention of late PPFFs.

## Introduction

There is no eternal total hip arthroplasty (THA). With time since the primary procedure, every implant will eventually fail due to the pathological conditions (e.g., infection, fracture, dislocation) or even normal/expected tribological and biological processes, such as wear and loosening (1). The ultimate aim is to provide normal pain-free function for the rest of the patients’ lives (2). Despite patients’ expectations and reliance on surgeons and implants to provide a timeless solution for their conditions, with varying perceptions of the required self-engagement, the surgery is, in fact, only the beginning. This is particularly true for the cementless THA concept, which relies, not only on early mechanical stability, but also on the host's potential to osseointegrate the implanted metallic components (3, 4).

Based on systematic reviews and meta-analysis, the risk of revision gradually increases in the third decade after the primary implantation, with estimates of three-quarters of THAs lasting at least 20 years, and only around 60% to 25 years (1, 5). With the mean age of patients undergoing THA being under 70 years, the female predominance, who have higher expected life span compared to males, and incrementally increasing overall life-expectancy reaching above 80 years, there are more and more implants at risk for failure (6, 7, 8). Among the four most common reasons for revision are infection, periprosthetic fractures (PPFs), loosening and dislocation. These four cumulatively constitutes for approximately 80% of complications (9, 10).

PPFs represent a complex orthopaedic pathology with significant patients’ morbidity and mortality, and socio-economic implications. In more than 80% of cases, the mechanism of injury is a low-energy trauma, mainly fall from the standing height. Most of the PPFs affect the proximal femur (PPFFs), while acetabulum is involved in less than 10% of THA-related fractures (11, 12).

PPFFs are associated with some already known patient- (age, female sex, osteoporosis/osteopenia, neuromuscular diseases, cognitive disorders, Paget's disease, developmental hip dysplasia, rheumatoid arthritis), surgical- (malposition, extensive broaching), and implant-related (cementless, design/type, loosening, stress shielding) risk factors (12–17). Interestingly, the research and developments have been for decades intensively focused mainly on the artificial implants, their materials, composition, design, and other characteristics, while the local host environmental factors have remained more unaddressed (18–22). However, at the end, it is the bone that fractures, which stimulated our efforts to develop a reliable and clinically applicable method for the evaluation of preserved proximal femoral bone stock volume around the implanted femoral stems (*V*_PF_) on routinely available hip radiographs.

The aim of the present study was to introduce and validate a novel method for the evaluation of *V_PF_* on widely available hip anterior-posterior (AP) radiographs, and to assess its influence on the risk of late PPFFs in conventional primary THA.

## Methods

### Method description: evaluation of the *V_PF_* parameter on hip AP radiographs

Geometrical parameters of the bone in contact with femoral stems were evaluated from the standard hip AP radiographs. Preferentially, the first postoperatively available standing radiographs were utilized. In the minority of cases (19), only the immediate postoperative radiographs on the operating table in supine position were available, and therefore had to be used for the *V_PF_* evaluation.

**Figure 1:**
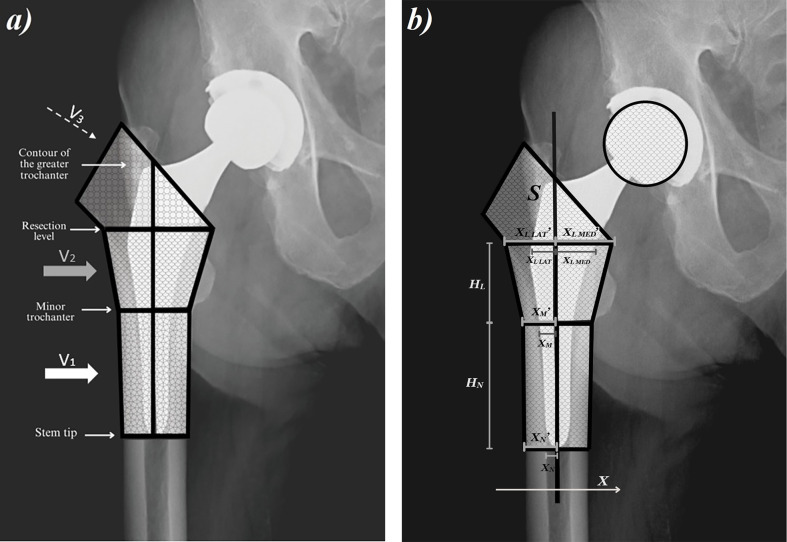
**(a)** segmentation of the femur in the model; **(b)** geometrical parameters for the *V_PF_* calculation. V, volume; H, height; S, surface; X, parameter.

The images were available in DICOM format and measured by software Agfa HealthCare Enterprise Imaging (Agfa-Gevaert NV, Mortsel, Belgium). This software enabled measurements of lengths and delimited areas. To calculate *V_PF_*, a mathematical model was constructed by composing parts of rotational bodies and a prism, subject to geometrical parameters of the proximal femur (the levels of femoral stem tip/smaller trochanter/femoral neck resection, and the contour of the greater trochanter) that were assessed from radiographs (Figure 1A). The parameters: X_N_’, X_M_’, X_L MED_’, X_L LAT_’, X_N_, X_M_, X_L MED_, X_L LAT_, H_N_, H_L_ and S, that were used for the calculations of respective volumes were presented in Figure 1B. The calculations were performed in Excel (Version 2016; Microsoft, Redmond, WA, USA). Since the magnifications of images were unknown, the dimensions of parameters were scaled utilizing the known diameters of prosthetic femoral heads. The in depth modelling technique and calculations have already been described in Kolar et al. study (23), and were summarized in the Appendix 1.

### Radiographic evaluation

Beside the already outlined main focus of the study (*V_PF_* parameter), hip radiographs were also employed for the evaluation of stress shielding, Canal Flare Index (CFI), Dorr classification, femoral neck resection level, and stem subsidence at different evaluation time points. For size calibration of all numeric parameters, the known diameters of prosthetic femoral heads were utilized.

Stress shielding, defined as a resorptive bone remodelling due to the alleviation of normal weightbearing stresses caused by the stiffness mismatch between the bone and implant, was assessed on the latest available postoperative hip radiographs (24). This phenomenon follows a fundamental principle of solid mechanics, where the stiffer material or structure carries the majority of the load, when the two materials are connected. The mean follow-up time of its radiographic evaluation was 60.8 ± 32.9 months. It was determined according to the Engh Grading Scale (25), which is a semiquantitative measure of bone resorption and as such inferior to the dual-energy x-ray absorptiometry (DEXA), which allows quantitative assessment of bone mineral density (BMD). The Engh Grading Scale classifies stress shielding into 5 grades: a) grade 0: absence of radiographic signs; b) grade 1: rounding of the proximal medial neck; c) grade 2: loss of the medial cortical density around the lesser trochanter; d) grade 3: more extensive loss of the medial cortical density expanding below the lesser trochanter; e) grade 4: cortical resorption into the diaphysis and all around the stem (25).

Canal Flare Index is a measure of proximal femoral canal morphology, defined as a quotient of medullary canal width two centimetres above the intertrochanteric line and the canal width at the isthmus (26). CFI may be interpreted as a numerical counterpart of the Dorr classification with 3 corresponding types of femoral morphology: a) Dorr type A (“champagne flute”): CFI ≥ 4.7; Dorr type B (“normal”): CFI 3.0–4.7; c) Dorr type C (“stovepipe”): CFI < 3.0 (27, 28). The CFI and Dorr type were both determined on the last preoperative radiographs.

The femoral neck resection level was measured as the vertical distance from the tip of the lesser trochanter to the bone resection cut on the medial side of the femoral neck. It was assessed on calibrated immediate postoperative radiographs.

Stem subsidence refers to the gradual axial displacement of the femoral component into the canal following the THA. It has been already proposed as an indirect measure of stem loosening/osseointegration. Subsidence was quantified by measuring the distance between the proximal aspect of the greater trochanter and the shoulder of the femoral stem, aligned parallel to the longitudinal axis of the stem (29, 30). The measurements were performed on calibrated radiographs at two postoperative time points: the first and last available radiographs. The subsidence was calculated as the difference between the two measurements. The mean follow-up time between the two measurements was 50.3 ± 30.1 months.

### Subjects

The retrospective matched case-control study was conducted according to the standard protocol for this type of research. Every case of PPFF, defined as a fracture occurring minimum 1 year postoperatively, was enrolled from the observational cohort of all implanted primary THA with a cementless ABG II (Anatomic Benoist Girard II, Stryker, Kalamazoo, MI, USA) femoral stem combined with either ABG II acetabular cup or acetabulum from another manufacturer, between January 1, 2012, and December 31, 2018, at a single tertiary hospital (University Medical Centre Ljubljana, Department of Orthopaedic Surgery, Ljubljana, Slovenia). All PPFFs of the cementless ABG II femoral stems up to December 31, 2023 were included. Clinical investigational plan was approved by the National Medical Ethics Committee (permit No. 0120-605/2021/3). For each case, 2 matched controls without the fracture as of December 31, 2023, were found from the whole observational cohort of 1531 cementless ABG II femoral stems. Matching was utilized for age, sex, implant size, and surgeon. None of the PPFFs cases and matched controls, who met the inclusion criteria, were excluded. In the group of cases, 30 patients with late PPFFs were identified. Correspondingly, the control group of 60 age-/sex-/impant size-/surgeon-matched patients was formed, and 30 matched stratums, each comprising a case and its 2 controls, were analysed. Patients’ demographics, medical history, stress shielding, CFI, Dorr type, femoral neck resection level, and stem subsidence were evaluated and documented (Table 1).

**Table 1:** Patients’ demographics, medical history and radiographic parameters in both groups.

Parameter	All (*n* = 90)	Cases (*n* = 30)	Controls (*n* = 60)	Comparison (*P* values)
Age (years)	73.1 ± 6.3	73.7 ± 6.3	72.9 ± 6.3	*P* = 0.57
Sex (*n*)				
Female	57 (63%)	19 (63%)	38 (63%)	*P* = 1.0
Male	33 (37%)	11 (37%)	22 (37%)	
Height (m)	166.6 ± 9.3	166.7 ± 8.5	166.5 ± 9.7	*P* = 0.90
Weight (kg)	77.1 ± 13.2	76.8 ± 13.2	77.3 ± 13.3	*P* = 0.87
BMI (kg/m^2^)	27.8 ± 4.3	27.6 ± 4.6	27.9 ± 4.2	*P* = 0.82
Level of femoral neck resection (mm)	14.9 ± 3.5	14.5 ± 3.7	15.2 ± 3.4	*P* = 0.38
Osteoporosis (*N*)	17 (19%)	5 (17%)	12 (20%)	*P* = 0.70
Stress shielding (Engh Grading Scale)	1.4 ± 0.9	2.1 ± 0.7	1.0 ± 0.8	*P* < 0.01
Subsidence (mm)	2.3 ± 1.5	3.8 ± 1.0	1.5 ± 0.9	*P* < 0.01
CFI	3.4 ± 0.8	2.9 ± 0.5	3.7 ± 0.8	*P* < 0.01
Dorr classification (A/B/C) (*N*)	12 (13%)/52 (58%)/26 (29%)	0 (0%)/16 (53%)/14 (47%)	12 (20%)/36 (60%)/12 (20%)	*P* < 0.01

CFI, canal flare index; BMI, body mass index; m, meter; mm, millimetre; kg, kilogram; kg/m^2^, kilogram per square meter.

Categorical variables were presented as frequencies (percentages), while continuous variables as means (standard deviation). For comparison of both groups Student *t* test (continuous variables) or Chi square test (categorical variables) were applied. The *P* values were reported.

### ABG II femoral stem

The cementless ABG II endoprosthesis provides femoral and acetabular components. It was introduced in 1996, as a successor of the ABG I, mostly to improve proximal stress transfer (31). The ABG II femoral stem was the most commonly used cementless stem option in Australia at the beginning of the 2000s, and extensively used in Europe (32). It was available in eight left and right sizes. The stem was made of titanium TMZF alloy (Titanium, Molybdenum, Zirconium, Ferrous), and was characterized as an anatomical type 6, based on Khanuja et al. classification, with 12° of built in anteversion (33, 34). The design goal was to obtain a close contact between the stem and bone in the proximal metaphyseal region, while the distal portion had an undersized and polished surface to avoid load transfer and bone ongrowth. The hydroxyapatite (HA) coating was applied by torch plasma spray to the proximal one-third of the stem. The Morse taper had an angle of 5°40’ (V40) and can be used with either a cobalt-chromium or alumina ceramic femoral heads. It could be combined with the hemispheric acetabular component that was made of titanium alloy (TiAl6V4) and fully coated with HA (34).

### Surgical intervention

Patients were operated under spinal or general anaesthesia, in the supine position with the direct lateral approach, or in the lateral decubitus position with the posterior approach to the hip joint. The cementless ABG II femoral stems were combined with either acetabular cup ABG II or acetabulum from another manufacturer. All surgical procedures were performed in the two operating rooms of the same operating suite of a single tertiary university hospital. Perioperative antibiotic prophylaxis, thromboembolic prophylaxis and postoperative rehabilitation protocol were uniform for all patients at a given time point, but they have been changing between 2012 and 2018 in accordance with the national guidelines.

### Statistics

Descriptive statistical analysis was used to assess patients’ demographics, medical history, stress shielding, CFI, resection level and subsidence. Continuous variables were presented as means with standard deviations (SD), and categorical variables as frequencies with corresponding percentages. For comparison of both groups either Student t test (continuous variables) or Chi square test (categorical variables) were applied. The accuracy of the method and the cut-off value of *V_PF_* were evaluated with the receiver operating characteristic (ROC) curve analysis determining the area under the curve (AUC) and utilizing Youden index for optimal *V_PF_* cut-off value. Conditional logistic regression model with the Cox regression analysis was utilized to assess the impact of covariables on the risk for PPFFs. The input covariables included: body mass index (BMI), osteoporosis, stress shielding, CFI, Dorr type, femoral neck resection level, subsidence, and the newly proposed *V_PF_*. Multivariable linear regression model was employed to analyse potential correlations between the *V_PF_* and basic demographics (age, sex, BMI), implant size, osteoporosis, stress shielding, CFI, Dorr type**,** femoral neck resection level, and stem subsidence. In the regression analysis, the categorical variables were managed with the transformation to dummy variables. Osteoporosis was determined if specific medications for its treatment were included in the patients’ regular therapy. Intra- and inter-rater reliability of the proposed method was tested and intraclass correlation coefficient (ICC) was calculated. Intra-rater ICC was measured on 45 randomly selected radiographs, inter-rater ICC between the junior (MK) and senior (BM) co-authors on 20, while the absolute agreement between 6 biophysics students and one of the co-authors (VKI) on 7 random standing hip radiographs. Also, the differences between the *V_PF_* measurements on standing and immediate postoperative (supine position on the operating table) hip radiographs were compared, and the ICC was calculated for 20 randomly selected cases who had both radiographs in the system. Statistical analysis was performed with SPSS (Version 25.0; IBM, Chicago, IL, USA). The level of statistical significance was set at *P* < 0.05.

## Results

The mean value of *V_PF_* in the group of cases was 113.8 ± 21.0 cm^3^ and significantly lower compared to the mean value of 164.0 ± 38.4 cm^3^ in the control group (*P* < 0.01). In only 1 out of 30 age-/sex-/implant size-/surgeon-matched strata, the mean *V_PF_* of both controls was lower than *V_PF_* of the case. As shown in Table 1, the significant differences were observed between both groups for the following radiographic parameters: stress shielding, subsidence, CFI and Dorr type (*P* < 0.01). In contrast, the basic demographic parameters, some of them were also utilized for matching, were well above the level of significance: age (*P* = 0.57), height (*P* = 0.90), weight (*P* = 0.87), BMI (*P* = 0.82), sex (*P* = 1.0). Also, there were no significant differences in the prevalence of osteoporosis (*P* = 0.70) and the levels of femoral neck resection (*P* = 0.38).

In the ROC curve analysis, the AUC was 0.90 (95% confidence interval: 0.83–0.96). Based on the Youden index, the optimal cut-off value of *V_PF_* was determined at 128.5 cm^3^.

The conditional logistic regression subanalysis of covariates potentially affecting the risk of PPFFs identified *V_PF_* as the sole parameter influencing this risk, with a hazard ratio of 0.92 for each additional cubic centimetre of *V_PF_* (95% confidence interval: 0.87–0.97; *P* < 0.01).

In the multivariable regression model for factors correlating with the *V_PF_*, the level of stress shielding, subsidence and sex turned out as significant. The results of both regression analyses were presented in Table 2.

**Table 2:** Results of conditional logistic and multivariable linear regression models on factors influencing the risk of late PPFFs and parameters correlating with the *V_PF_*.

Dependent variable	Significant predictors	B	*P*-value	aOR
PPFFs risk	*V_PF_*	−.08	<0.01	0.92
Dependent variable	Significant predictors	B	*P*-value	
*V_PF_*	*Stress shielding*	−.28	<0.01	
	*Subsidence*	−.35	<0.01	
	*Sex*	.40	<0.01	

*V_PF_*, volume of preserved proximal femoral bone stock; PPFFs, periprosthetic proximal femoral fractures; aOR, adjusted Odds Ratio; B, regression coefficient.

Only statistically significant independent covariables are reported (*P* < .05). Sex was coded: Female = 1, Male = 2; Osteoporosis: No = 0, Yes = 1.

The ICC values for intra- and inter-rater reliabilities, as well as for the comparison of *V_PF_* measurements in standing and supine positions, along with the interpretation of agreement levels were reported in Table 3.

**Table 3:** Reliability testing of V_PF_ measurements using ICC.

Reliability type	ICC value	95% CI	Agreement
Intra-rater	0.96	0.93–0.98	Excellent
Inter-rater (Junior vs. Senior)	0.83	0.60–0.93	Good
Inter-rater(students and senior biophysicist)	0.82	0.52–0.96	Good
Standing vs. supine	0.91	0.80–0.96	Excellent

ICC, intraclass correlation coefficient; CI, confidence interval.

The agreement levels for intra- and inter-rater reliability, as well as between standing and supine hip radiographs were reported with 95% confidence intervals for ICC values.

## Discussion

The present study introduced a novel measure of preserved proximal femoral bone stock around the implanted femoral stems and the method for its evaluation on the routinely available hip radiographs. The main finding was the validated importance of bone stock preservation in reducing the risk of late PPFFs, based on significantly lower V_PF_ values in fractured proximal femora around ABG II stems compared to unfractured control subjects with identical implants of the same size, as well as the results of the regression subanalysis. The difference in preserved bone stock was not only significant, but also substantial.

Despite the longstanding awareness of the increasing incidence and consequences of PPFFs, and the rationale protective role of the preserved bone stock, no method for its evaluation, with the potential for routine clinical application, has been available. The introduced method and *V_PF_* parameter represent a useful tool for the assessment of future PPFFs risk. One of the main advantages of this newly developed method is in its routine and wide availability based on the possibility to utilize standard hip radiographs, which are part of every routine diagnostic evaluation of patients with the indication for primary THA. Moreover, its simplicity, quick learning curve, and time efficiency (taking only a few minutes after some examples measured) enable the surgeon to plan and control the bone stock preservation to some degree for every patient. Also, the intra- and inter-rater reliability demonstrated good to excellent levels of agreement.

Based on the ROC curve analysis with the AUC value of 0.90, the proposed method also demonstrated good to excellent performance with the lowest end of 95% IC interval being 0.83 and above the 0.80 mark for good level of accuracy (35). The cut-off value of *V_PF_* was determined with Youden index at 128.5 cm^3^, under which the consideration of different approach is advisable. This may include the use of cemented femoral stem at THA or other classes of cementless femoral stems, beside the anatomical type 6 (Khanuja et al. classification), which potentially allow greater bone preservation (33). So far, the conventional stems were being analysed, while the effect of using short femoral stems with their potential to preserve more bone stock and provide better physiological load transfer will show consequences for PPPFs risk in the future studies (36).

The proposed method was developed on the cohort of patients with PPFFs of ABG II femoral stems, for which clinical results have been extensively reported. Previous studies of this implant encompassed arthroplasty registry data, multiple centres and single hospital reports (31, 32, 34, 37–42). Most of them documented satisfactory results with survival rates until the first revision in the 94%–100% range at approximately 6 years (32, 38), 89%–98% range at around 10 years (31, 34, 39), and even up to 96.1% at 14 years of follow-up (40). However, in many studies the increased risk of periprosthetic fractures was observed with notable differences in the revision profiles between the ABG II and all other conventional cementless primary THAs. In some reports, the PPFFs accounted for nearly or even more than half causes for revision (32, 37, 39, 43).

Many factors have been associated with increased risk of PPFFs (12, 13, 17, 19). Especially with time since the implantation, the interactions between the implant and the local host bone seem to be crucial. This relationship appears to depend on the conditions set at the time of implantation, which impact the long-term interactions. In cementless technique, the femoral morphology, implant selection, their compatibility allowing correct stem orientation, fill, stability and early fixation, as well as the newly proposed *V_PF_* parameter, seem to influence the two main long-term processes of osseointegration and stress shielding associated with the risk of PPFFs (3, 21, 44, 45). Based on the results of the present study, the *V_PF_* appears to be at the centre of interplay of these interconnected factors. Therefore, in establishing causal relationships between various parameters and PPFFs, the preserved bone stock could represent an unrecognized confounder.

In the regression model for determining the factors that significantly impact the risk of PPFFs, the *V_PF_* turned out as the sole statistically significant independent predictor after adjustment for age, sex, implant size and surgeon. With every preserved cubic centimetre of bone stock, the risk of PPFFs reduced for approximately 8%. Since the diagnosis of osteoporosis was also included in the model, it may be even speculated that the volume of preserved bone seems to be more vital than its quality. In addition to the positive correlation of *V_PF_* with the male sex, the significant inverse correlations of late PPFFs risk with the two crucial long-term factors, stress shielding and subsidence, were observed. The exact protective mechanism of preserved bone stock is still a ‘black box’, however it may be proposed as a common biological denominator of these parameters at interplay. It appears to support initial stability and enhance osseointegration acutely, while reducing stress shielding and preventing loosening (ensuring stable osseointegration without subsidence) in the long-term. Moreover, the difference between both groups in the indicators of proximal femoral canal morphology (CFI, Dorr type) was significant. That is in line with the established increased risk of late PPFFs in the Dorr type C femurs (45, 46). It may be even hypothesized that lower CFI, which is a numerical counterpart to the Dorr type C, reduces the chance of implantation with sufficient *V_PF_* preservation. Therefore, the bone stock preservation must be considered, starting from the preoperative planning onwards.

Several limitations of the present study should be acknowledged. It employed a retrospective design and included only one type of femoral stem. Further multi-centre studies including different stem types and their comparisons are needed prior to broad generalisation of this new concept. Key parameters (age, sex, implant size, surgeon) were controlled for, and a broad set of established risk factors was included in the analysis; however, residual confounding cannot be completely excluded in a real clinical setting. Some parameters were semiquantitative (stress shielding) or indirectly assessed (osteoporosis). Several measurements were performed on supine radiographs, and the rotational as well as parallax effects were not specially accounted for. The novel method was not yet validated by comparison with other methods for assessment of the risk for periprosthetic fractures. However, based on the reliability testing, not only intra- and inter-observer but also between standing and supine radiographs, the agreements were in the good to excellent range. Moreover, the ROC analysis demonstrated adequate performance of the novel method, and the overall results emphasized the importance of bone stock preservation with the introduced *V_PF_* parameter as its measure.

## Conclusions

We introduced a simple and widely accessible method to assess the risk for periprosthetic femoral fractures in THA. The method was tested on two populations: hips that sustained periprosthetic fractures and unfractured hips, demonstrating a considerable and statistically significant difference between the two groups. Reliability testing, including intra- and inter-observer assessments and agreement between supine and standing views was good to excellent. Further validation is needed to develop a protocol that can be used in clinical practice, however, the presented results indicate that bone stock preservation (with *V_PF_* as a quantitative measure) is crucial for the prevention of late PPFFs.

## Data Availability

The raw data supporting the conclusions of this article will be made available by the authors, without undue reservation.

## Appendix 1. Calculation of the *V_PF_* parameter.

*V_PF_* is composed of a hollow truncated cone (representing bone of the femoral shaft in contact with the femoral stem) and a prism (representing remnants of the greater trochanter). In general, the volume of a conus with base radius R and height H is given by.

$$V_{conus}=\pi R^{2}H/3,$$
(1)

and the volume of the truncated conus cut at height h with the radius being r is

$$V_{truncated\_conus}=\pi h\;(R^{2}+\;Rr\;+\;r^{2})/3,$$
(2)

where

r=R(1−h/H).
(3)

Following the Equation 2, at the lower part of the shaft, between the stem tip and minor trochanter (Figure 1A, white arrow), the bone is taken as symmetric with respect to the centreline, and the volume of the hollow truncated cone *V_1_* is

$$V_{1}=\pi (X_{N}\prime^{2}H_{N}+\;X_{N}\prime \;X_{M}\prime \;H_{N}+\;X_{M}\prime^{2}H_{N})/3\;-\pi (X_{N}^{2}H_{N}+\;X_{N}X_{M}H_{N}+\;X_{M}^{2}H_{N})/3.$$
(4)

In the middle part, between the minor trochanter and the resection level (Figure 1A, grey arrow), the bone contour is considered asymmetric relative to the central axis. Geometrical parameters for the lateral and medial sides are assessed separately. Volume of a rotational body is calculated for each set of parameters, with half of the total volume *V_2_*assigned to each side (*V_2 MED_*, *V*_2 LAT_),

$$V_{2\;\mathrm{MED}}=\pi (X_{L\;MED}\prime^{2}H_{L}+X_{L\;MED}\prime X_{M}\prime H_{L}+X_{M}\prime^{2}H_{L})/6\;-\pi (X_{L\;MED}^{2}H_{L}+\;X_{L\;MED}X_{M}H_{L}+X_{M}^{2}H_{L})/6,$$
(5)

$$V_{2\;\mathrm{LAT}}=\pi (X_{L\;LAT}\prime^{2}H_{L}+X_{L\;LAT}\prime X_{M}\prime H_{L}+X_{M}\prime^{2}H_{L})/6\;-\pi (X_{L\;LAT}^{2}H_{L}+X_{L\;LAT}X_{M}H_{L}+X_{M}^{2}H_{L})/6.$$
(6)

The volume of the bone remnants at the greater trochanter *V*_3_ (Figure 1A, dashed white arrow) is approximated by the volume of a prism, with the surface of the bone contour *S* and thickness of the femur *X*_N_’,

$$V_{3}=\;2\;SX_{N}\prime .$$
(7)

To obtain the volume of bone in contact with the femoral stem, the contributions of all parts are summed up,

$$V_{PF}=V_{1}+V_{2\;\mathrm{MED}}+V_{2\;\mathrm{LAT}}+V_{3.}$$
(8)

## References

[1] Evans JT Evans JP Walker RW Blom AW Whitehouse MR Sayers A. How long does a hip replacement last? A systematic review and meta-analysis of case series and national registry reports with more than 15 years of follow-up. Lancet. (2019) 393(10172):647–54. 10.1016/S0140-6736(18)31665-9 30782340 PMC6376618

[2] Karachalios T Komnos G Koutalos A. Total hip arthroplasty: survival and modes of failure. EFORT Open Rev. (2018) 3(5):232–9. 10.1302/2058-5241.3.170068 29951261 PMC5994632

[3] Sumner DR. Long-term implant fixation and stress-shielding in total hip replacement. J Biomech. (2015) 48(5):797–800. 10.1016/j.jbiomech.2014.12.021 25579990

[4] Konan S Abdel MP Haddad FS. Cemented versus uncemented hip implant fixation: should there be age thresholds? Bone Joint Res. (2020) 8(12):604–7. 10.1302/2046-3758.812.BJR-2019-0337 31934332 PMC6946910

[5] Ferguson RJ Palmer AJ Taylor A Porter ML Malchau H Glyn-Jones S. Hip replacement. Lancet. (2018) 392(10158):1662–71. 10.1016/S0140-6736(18)31777-X 30496081

[6] Schwartz AM Farley KX Guild GN Bradbury TL Jr. Projections and epidemiology of revision hip and knee arthroplasty in the United States to 2030. J Arthroplasty. (2020) 35(6S):S79–85. 10.1016/j.arth.2020.02.030 32151524 PMC7239745

[7] Santiago MS Doria FM Morais Sirqueira Neto J Fontes FF Porto ES Aidar FJ Platelet-rich plasma with versus without hyaluronic acid for hip osteoarthritis: a systematic review and meta-analysis. Front Bioeng Biotechnol. (2025) 13:1545431. 10.3389/fbioe.2025.1545431 40206824 PMC11980421

[8] Alahmari KA Reddy RS. Biomechanical analysis of limits of stability using computerized posturography: correlations with functional mobility in elderly individuals with hip osteoarthritis—a cross-sectional study. Front Bioeng Biotechnol. (2024) 12:1440393. 10.3389/fbioe.2024.1440393 39654827 PMC11625571

[9] Smith PN Gill DR McAuliffe MJ McDougall C Stoney JD Vertullo CJ Hip, knee and shoulder arthroplasty: 2023 annual report, Australian orthopaedic association national joint replacement registry, AOA: Adelaide, South Australia. (2023). 10.25310/YWQZ9375. https://aoanjrr.sahmri.com/documents/10180/1579982/AOA_NJRR_AR23.pdf

[10] Guo S Zhang J Li H Zhang J Cheng CK. A multi-branch network to detect post-operative complications following hip arthroplasty on x-ray images. Front Bioeng Biotechnol. (2023) 11:1239637. 10.3389/fbioe.2023.1239 37840662 PMC10569301

[11] Abdel MP Cottino U Mabry TM. Management of periprosthetic femoral fractures following total hip arthroplasty: a review. Int Orthop. (2015) 39:2005–10. 10.1007/s00264-015-2979-0 26318883

[12] Patsiogiannis N Kanakaris NK Giannoudis PV. Periprosthetic hip fractures: an update into their management and clinical outcomes. EFORT Open Rev. (2021) 6:75–92. 10.1302/2058-5241.6.200050 33532088 PMC7845569

[13] Luger M Feldler S Schopper C Gotterbarm T Stadler C. Is there a difference in pelvic and femoral morphology in early periprosthetic femoral fracture in cementless short stem total hip arthroplasty via an anterolateral approach? J Orthop Traumatol. (2024) 25(1):51. 10.1186/s10195-024-00795-x 39495408 PMC11535139

[14] Zheng S Zhu J Chen Z Cao X Xia T Zhang C AI-assisted direct anterior approach versus posterolateral approach in total hip arthroplasty: a retrospective cohort study based on artifact-reduced CT 3D reconstruction. Front Bioeng Biotechnol. (2025) 13:1509200. 10.3389/fbioe.2025.1509200 40297284 PMC12035441

[15] Gielen AMC Leijten NM Balraadjsing PPS Braakhuis HM Abee H Arts JJ Utilizing biomaterial surface properties to improve orthopedic hip implant safety and function in a safe-by-design approach. Front Bioeng Biotechnol. (2025) 13:1504883. 10.3389/fbioe.2025.1504883 40059888 PMC11885263

[16] Wang Z Wang Z Gu L Zhang Y Su T Luo J 3D-printed porous tantalum for acetabular reconstruction in complex primary arthroplasty and revision of hip. Front Bioeng Biotechnol. (2025) 13:1557882. 10.3389/fbioe.2025.1557882 40497252 PMC12149209

[17] Berend ME Smith A Meding JB Ritter MA Lynch T Davis K. Long-term outcome and risk factors of proximal femoral fracture in uncemented and cemented total hip arthroplasty in 2551 hips. J Arthroplasty. (2006) 21(6 Suppl 2):53–9. 10.1016/j.arth.2006.05.014 16950062

[18] Burchard R Graw JA Soost C Schmitt J. Stress shielding effect after total hip arthroplasty varies between combinations of stem design and stiffness-a comparing biomechanical finite element analysis. Int Orthop. (2023) 47:1981–7. 10.1007/s00264-023-05825-7 37269400 PMC10345085

[19] Carli AV Negus JJ Haddad FS. Periprosthetic femoral fractures and trying to avoid them: what is the contribution of femoral component design to the increased risk of periprosthetic femoral fracture? Bone Joint J. (2017) 99-B(1 Supple A):50–9. 10.1302/0301-620X.99B1.BJJ-2016-0220.R1 28042119

[20] Rivière C Grappiolo G Engh CA Jr Vidalain JP Chen AF Boehler N Long-term bone remodelling around ‘legendary’ cementless femoral stems. EFORT Open Rev. (2018) 3:45–57. 10.1302/2058-5241.3.170024 29657845 PMC5890130

[21] Glassman AH Bobyn JD Tanzer M. New femoral designs: do they influence stress shielding? Clin Orthop Relat Res. (2006) 453:64–74. 10.1097/01.blo.0000246541.41951.20 17312586

[22] Hirata Y Inaba Y Kobayashi N Ike H Fujimaki H Saito T. Comparison of mechanical stress and change in bone mineral density between two types of femoral implant using finite element analysis. J Arthroplasty. (2013) 28(10):1731–5. 10.1016/j.arth.2013.04.034 23683518

[23] Kolar M Mavčič B KraljIglič V Antolič V. Preserved proximal femoral bone stock volume in total hip arthroplasty significantly reduces the risk for periprosthetic fractures. a novel modelling technique and preliminary clinical results. Proc Socratic Lect. (2024) 10:13–21. 10.55295/PSL.2024.I3

[24] Huiskes R Weinans H Grootenboer HJ Dalstra M Fudala B Slooff TJ. Adaptive bone-remodeling theory applied to prosthetic-design analysis. J Biomech. (1987) 20(11-12):1135–50. 10.1016/0021-9290(87)90030-3 3429459

[25] Engh C Bobyn J Glassman A. Porous-coated hip replacement. The factors governing bone ingrowth, stress shielding, and clinical results. J Bone Joint Surg Br. (1987) 69-B(1):45–55. 10.1302/0301-620X.69B1.3818732 3818732

[26] Noble PC Alexander JW Lindahl LJ Yew DT Granberry WM Tullos HS. The anatomic basis of femoral component design. Clin Orthop Relat Res. (1988) 235:148–65. 10.1097/00003086-198810000-00015 3416522

[27] Dorr LD Faugere MC Mackel AM Gruen TA Bognar B Malluche HH. Structural and cellular assessment of bone quality of proximal femur. Bone. (1993) 14:231–42. 10.1016/8756-3282(93)90146-2 8363862

[28] Wilkerson J Fernando ND. Classifications in brief: the dorr classification of femoral bone. Clin Orthop Relat Res. (2020) 478(8):1939–44. 10.1097/CORR.0000000000001295 32732579 PMC7371079

[29] Bornes TD Radomski LR Bonello JP Mortensen-Truscott L Safir OA Gross AE Subsidence of a single-taper femoral stem in primary total hip arthroplasty: characterization, associated factors, and sequelae. J Arthroplasty. (2023) 38(7S):S174–8. 10.1016/j.arth.2023.04.026 37088226

[30] Streit MR Haeussler D Bruckner T Proctor T Innmann MM Merle C Early migration predicts aseptic loosening of cementless femoral stems: a long-term study. Clin Orthop Relat Res. (2016) 474(7):1697–706. 10.1007/s11999-016-4857-5 27130649 PMC4887381

[31] Herrera A Mateo J Lobo-Escolar A Panisello JJ Ibarz E Gracia L. Long-term outcomes of a new model of anatomical hydroxyapatite-coated hip prosthesis. J Arthroplasty. (2013) 28(7):1160–6. 10.1016/j.arth.2012.06.033 23134598

[32] Catanach MJ Sorial RM Eslick GD. Thirteen-year outcomes in the anatomique benoist girard II hip prosthesis. ANZ J Surg. (2015) 85(4):255–9. 10.1111/ans.12894 25367866

[33] Khanuja HS Vakil JJ Goddard MS Mont MA. Cementless femoral fixation in total hip arthroplasty. J Bone Joint Surg Am. (2011) 93(5):500–9. 10.2106/JBJS.J.00774 21368083

[34] Nourissat C Essig J Asencio G. The cementless anatomic benoist girard (ABG) II total hip arthroplasty: a minimum 8-year follow-up study. J Arthroplasty. (2013) 28(4):707–11. 10.1016/j.arth.2012.07.022 23122655

[35] Koo TK Li MY. A guideline of selecting and reporting intraclass correlation coefficients for reliability research. J Chiropr Med. (2016) 15:155–63. 10.1016/j.jcm.2016.02.012 27330520 PMC4913118

[36] Anderl C Steinmair M Hochreiter J. Bone preservation in total hip arthroplasty. J Arthroplasty. (2022) 37(6):1118–23. 10.1016/j.arth.2022.01.077 35121089

[37] Mulford JS Mathew R Penn D Cuthbert AR de Steiger R. Periprosthetic fracture as a late mode of failure of the anatomique benoist girard II femoral prosthesis. ANZ J Surg. (2022) 92:1165–70. 10.1111/ANS.17547 35191171 PMC9306843

[38] Rogers A Kulkarni R Downes EM. The ABG hydroxyapatite-coated hip prosthesis: one hundred consecutive operations with average 6-year follow-up. J Arthroplasty. (2003) 18(5):619–25. 10.1016/s0883-5403(03)00208-0 12934215

[39] Kropivšek L Antolič V Mavčič B. Surgeon-stratified periprosthetic fracture risk in a single-hospital cohort of 1531 uncemented ABG-II femoral stems at primary total hip arthroplasty. Indian J Orthop. (2023) 57(11):1850–7. 10.1007/s43465-023-00996-2 37881273 PMC10593654

[40] Epinette JA Asencio G Essig J Llagonne B Nourissat C. Clinical results, radiological fndings and survival of a proximally hydroxyapatite-coated hip ABG II stem at a minimum of ten years’ follow-up: results of a consecutive multicentre study of 1148 hips in 1053 patients. Bone Joint Journal. (2013) 95:1610–6. 10.1302/0301-620X.95B12 24293589

[41] Van der Wal BC Rahmy AI Grimm B Blake GM Heyligers IC Tonino AJ. The influence of implant design on periprosthetic bone remodelling of two types of uncemented HA-coated hip stems. A two-year follow-up study using DEXA. Hip Int. (2006) 16(1):8–17. 10.5301/hip.2008.971 19219772

[42] Coulomb R Mansour J Essig J Asencio G Kouyoumdjian P. Clinical results at 10 years of minimum follow-up with the ABG 2 hip arthroplasty, matched with ceramic-on-ceramic bearings. SICOT J. (2022) 8:32. 10.1051/sicotj/2022032 35969123 PMC9377216

[43] Thien TM Chatziagorou G Garellick G Furnes O Havelin LI Mäkelä K Periprosthetic femoral fracture within two years after total hip replacement: analysis of 437,629 operations in the nordic arthroplasty register association database. J Bone Joint Surg Am. (2014) 96(19):e167. 10.2106/JBJS.M.00643 25274795

[44] Savio D Bagno A. When the total hip replacement fails: a review on the stress-shielding effect. Processes. (2022) 10:612. 10.3390/pr10030612

[45] Bigart KC Nahhas CR Ruzich GP Culvern CN Salzano MB Della Valle CJ Does femoral morphology predict the risk of periprosthetic fracture after cementless total hip arthroplasty? J Arthroplasty. (2020) 35(6S):S359–63. 10.1016/j.arth.2020.02.048 32209287

[46] Kheir MM Dilley JE Speybroeck J Kuyl EV Ochenjele G McLawhorn AS The influence of dorr type and femoral fixation on outcomes following total hip arthroplasty for acute femoral neck fractures: a multicenter study. J Arthroplasty. (2023) 38(4):719–25. 10.1016/j.arth.2022.10.028 36283515

